# Effects of endplate coverage and intervertebral height change on heterotopic ossification following cervical disc replacement

**DOI:** 10.1186/s13018-021-02840-5

**Published:** 2021-11-25

**Authors:** Yi-Wei Shen, Yi Yang, Hao Liu, Xin Rong, Chen Ding, Yang Meng, Bei-Yu Wang, Ying Hong

**Affiliations:** 1 Department of Orthopedic Surgery, West China Hospital, Sichuan University, No. 37 Guo Xue Rd, Chengdu, 610041 China; 2 Department of Operation Room and Anesthesia Center, West China Hospital, Sichuan University, No. 37 Guo Xue Rd, Chengdu, 610041 China

**Keywords:** Cervical disc replacement, Heterotopic ossification, Endplate coverage, Intervertebral height change

## Abstract

**Background:**

Heterotopic ossification (HO) is a common complication after cervical disc replacement (CDR). Biomechanical factors including endplate coverage and intervertebral disc height change may be related to HO formation. However, there is a dearth of quantitative analysis for endplate coverage, intervertebral height change and their combined effects on HO.

**Methods:**

Patients who underwent single-level or two-level CDR with Prestige-LP were retrospectively reviewed. Clinical outcomes were evaluated through Japanese Orthopaedic Association (JOA) score, Neck Disability Index (NDI) score, and visual analogue scale (VAS) score. Radiological data, including the prosthesis-endplate depth ratio, intervertebral height change, posterior heterotopic ossification (PHO) and angular parameters, were collected. Logistic regression analysis was used to identify the potential risk factors. Receiver operating characteristic curves were plotted and the cut-off values of each potential factors were calculated.

**Results:**

A total of 138 patients with 174 surgical segments were evaluated. Both the prosthesis-endplate depth ratio (*P* < 0.001) and post-operative disc height change (*P* < 0.001) were predictive factors for PHO formation. The area under the curve (AUC) of the prosthesis-endplate depth ratio, disc height change and their combined effects represented by the combined parameter (CP) were 0.728, 0.712 and 0.793, respectively. The risk of PHO significantly increased when the prosthesis-endplate depth ratio < 93.77% (*P* < 0.001, OR = 6.909, 95% CI 3.521–13.557), the intervertebral height change ≥ 1.8 mm (*P* < 0.001, OR = 5.303, 95% CI 2.592–10.849), or the CP representing the combined effect < 84.88 (*P* < 0.001, OR = 10.879, 95% CI 5.142–23.019).

**Conclusions:**

Inadequate endplate coverage and excessive change of intervertebral height are both potential risk factors for the PHO after CDR. Endplate coverage less than 93.8% or intervertebral height change more than 1.8 mm would increase the risk of PHO. The combination of these two factors may exacerbate the non-uniform distribution of stress in the bone-implant interface and promote HO development.

## Introduction

Anterior cervical discectomy and fusion (ACDF) has been the standard procedure concerning surgical treatment for cervical myelopathy or radiculopathy for several decades. Although advances in implants and surgical techniques of ACDF have led to better outcomes and fewer complications, the fusion procedure sacrifices the segmental mobility and alters the biomechanical status of adjacent intervertebral discs, which may accelerate adjacent segmental pathology with or without symptoms [1]. Cervical disc replacement (CDR) is an effective option for patients diagnosed as cervical myelopathy or radiculopathy. Previous randomized controlled studies with long-term results have demonstrated that CDR has at least equivalent clinical outcomes compared to ACDF, with lower incidence of adjacent segment disease (ASD) [2–5]. However, heterotopic ossification (HO), also known as paravertebral ossification or post-operative bone formation, occurred as one of the major complications of CDR [6–8]. HO is considered an intractable issue probably resulting in the loss of segmental mobility and poor neurological recovery [9, 10].

The formation of HO following CDR is multifactorial, including the preoperative ossification, surgical technique and biomechanical elements [11, 12]. The change of biomechanical environment of the surgical segment caused by artificial disc implantation is one of the major considerations of HO formation. Ganbat et al. [13] found that HO formation might play a role in compensating for the non-uniform stress distribution of prosthesis-endplate interface after CDR. Biomechanical factors, including endplate coverage and disc height, may play roles in this process [14]. Prior studies suggested that insufficient endplate coverage could lead to the occurrence of HO [15–17]. However, due to the fixed size of prosthesis and the irregular morphology of cervical endplate, the endplate coverage may not be always optimal during operation. There is a dearth of quantitative analysis for the relationship between endplate coverage and HO occurrence for intraoperative reference. Besides, Kim et al. [18] found that over-distraction of surgical segment and increase in the segmental mobility would affect the HO formation. Inappropriate increment of intervertebral height would further increase the stress of prosthesis-endplate interface in the scenario of insufficient endplate coverage [19]. However, studies about the combined effects of endplate coverage and disc height change on HO formation are still scarce. Several retrospective studies reported that insufficient coverage of endplate may induce HO formation while the effect of intervertebral disc height change was not analysed [12, 15, 16, 20]. Additionally, other studies emphasized the effect of disc height on HO formation while the endplate coverage was not scrutinized [21, 22]. Therefore, this study aimed to investigate the effects of endplate coverage and intervertebral disc height change post-operatively and the combination of these two factors on HO formation following CDR through quantitative analysis.

## Methods

### Patient population

This retrospective study included patients who underwent 1-level and 2-level Prestige-LP CDR from January 2010 to January 2019 with a minimum of 2 years follow-up. The study protocol was approved by the Medical Ethical Committee of West China Hospital of Sichuan University and all patients provided written informed consent. Patients were included if they (1) were 18–65 years of age; (2) were diagnosed as 1-level or 2-level cervical degenerative disc disease causing symptomatic radiculopathy or myelopathy between C3 and C7; and (3) failed strict conservative therapy for at least 12 weeks. The exclusion criteria included: (1) instability, irreducible kyphosis, or severe degeneration at the surgical segment; (2) prior history of cervical spine surgery; (3) patients diagnosed as non-degenerative cervical spine diseases; (4) ossification of the posterior longitudinal ligament; (5) osteoporosis.

### Surgical procedure

The same senior spine surgeon treated all the patients. A standard right-side Smith-Robinson approach was performed after general anaesthesia. Complete discectomy and decompression were conducted at the index level by removing the anterior longitudinal ligament, disc tissue, posterior longitudinal ligament and osteophytes, followed by careful endplate preparation with a high-speed burr. Then, a rail cutter guide and bit were used to drill the fixation channels in the endplate, and an appropriate Prestige-LP disc was inserted into the indicated level. Proper placement of the prosthesis was verified by C-arm fluoroscopy. Copious irrigation with normal saline and meticulous haemostasis were conducted. The same procedure was performed at the other level in 2-level cases. Finally, the incision was sutured layer by layer after inserting a drainage tube. Nonsteroidal anti-inflammatory drugs were not routinely used for preventing HO in this cohort.

### Data collection and measurement

Clinical and radiological data were collected preoperatively, 1 week post-operatively and at last follow-up. Clinical outcomes were evaluated through Japanese Orthopaedic Association (JOA) score, Neck Disability Index (NDI) functional score, and visual analogue scale (VAS) score.

**Fig. 1:**
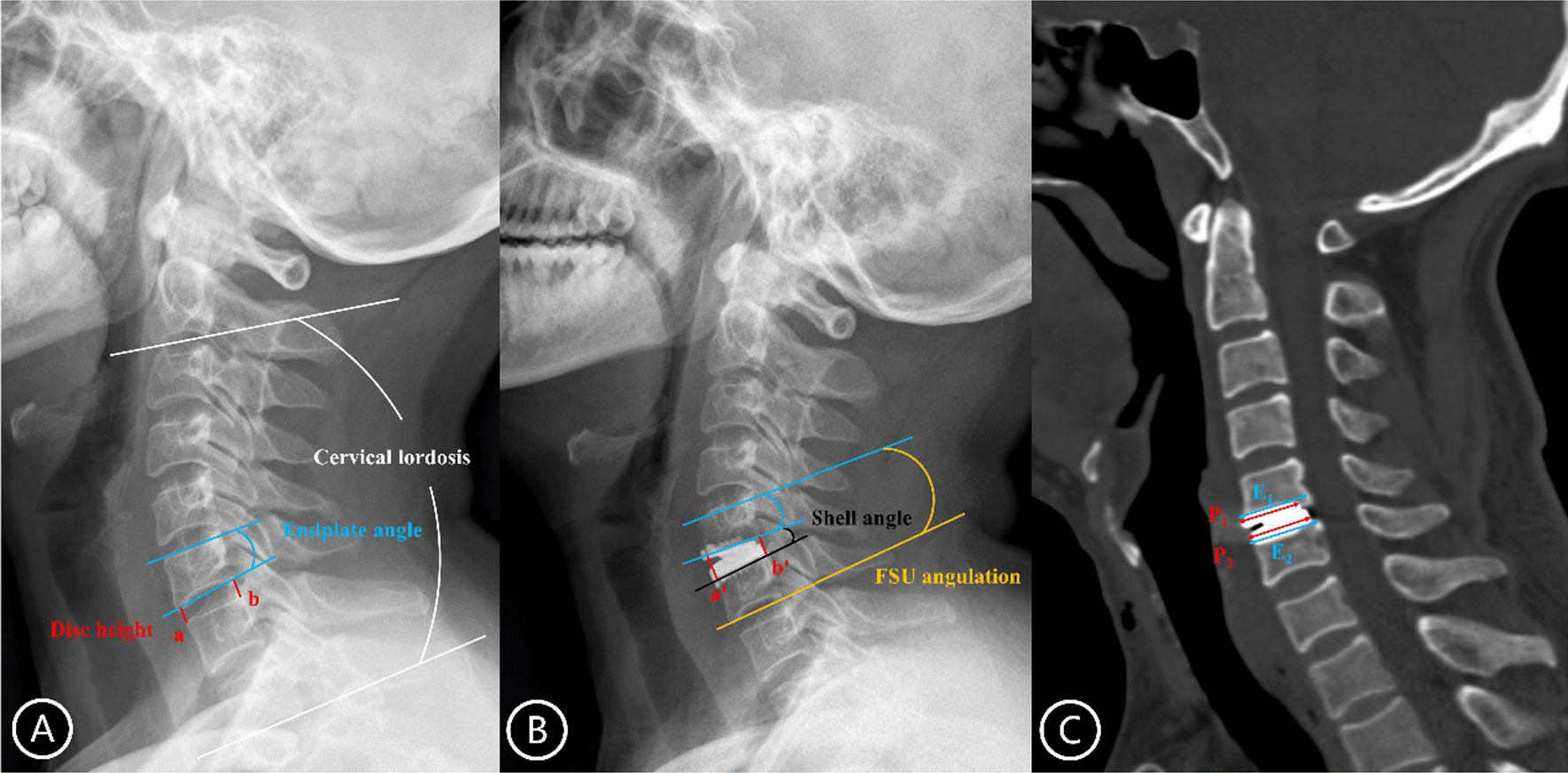
Measurement of radiological parameters. **A**, **B** Post-operative change of intervertebral disc height is calculated as (a’ + b’)/2 − (a + b)/2. Cervical lordosis is defined as the angle between the inferior endplate of C2 vertebra and the inferior endplate of C7 vertebra. Endplate angle of the cranial vertebra is the angle between the upper and lower endplates of the cranial vertebral body at index level. Shell angle is recorded as the angle between the superior and inferior endplate of the prosthesis. FSU angulation is the angle between the superior endplate of cranial vertebral body and the inferior endplate of the caudal vertebral body at the indicated segment. **C** Prosthesis-endplate depth ratio is calculated as (P_1_/E_1_ + P_2_/E_2_)/2. FSU, functional spinal unit

HO and anterior bone loss (ABL) were assessed on the lateral X-rays at the last follow-up. Due to the anterior-limiting design of Prestige-LP disc, inadequate coverage of endplate predominantly occurred in the posterior endplate region. Thus, only posterior heterotopic ossification (PHO) was assessed in this study. According to the McAfee classification, Grade 3–4 was classified as motion-restricting HO. ABL was defined as the reduction in subchondral vertebral body length during follow-up compared with the post-operative lateral radiograph as previous study described [23]. Disc height was measured at the lateral radiograph before and after surgery (Fig. 1a, b). The prosthesis-endplate depth ratio was calculated on the median sagittal plane of reconstruction computed tomography (CT) as dividing the length of the prosthesis by the immediate post-operative length of the endplate [15] (Fig. 1c). Cervical lordosis was the angle formed between the inferior endplate of C2 vertebra and the inferior endplate of C7 vertebra. C2–C7 range of motion (ROM) were measured on the flexion and extension radiographs using the Cobb method. Shell angle was defined as the angle drawn from the superior and inferior endplate of the prosthesis. Functional spinal unit (FSU) angulation was the angle between the lines of superior endplate of cranial vertebral body and the inferior endplate of the caudal vertebral body at the indicated segment. Endplate angle of the cranial vertebra was recorded as the angle between the upper and lower endplates of the cranial vertebral body at index level. The changes of endplate angle between pre-operation and 1 week post-operatively were defined as the milling angle. The change in disc insertion angle was defined as the difference of endplate angle between pre-operation and last follow-up, representing the degree to which the inserted prosthesis deviates from the natural disc position [24] (Fig. 1).

### Statistical analysis

All statistical analyses were performed using SPSS Version 25.0 software (IBM Corp., Armonk, New York, USA). The results were presented as mean ± standard deviation for continuous variables and number of cases for categorical variables. Comparison of parameters between pre- and post-operation was conducted using paired *t* test. The independent *t* test or the Mann–Whitney U test was used to compare continuous variables depending on the normality of data. The Chi-square or Fisher’s exact test was used for categorical variables. Potential risk factors with *P* < 0.05 or those with clinical significance were involved in the logistic regression analysis. Receiver operating characteristic (ROC) curves of each variable were calculated. A 2-tailed *P* values < 0.05 were considered statistically significant.

## Results

### Demographic data

**Fig. 2:**
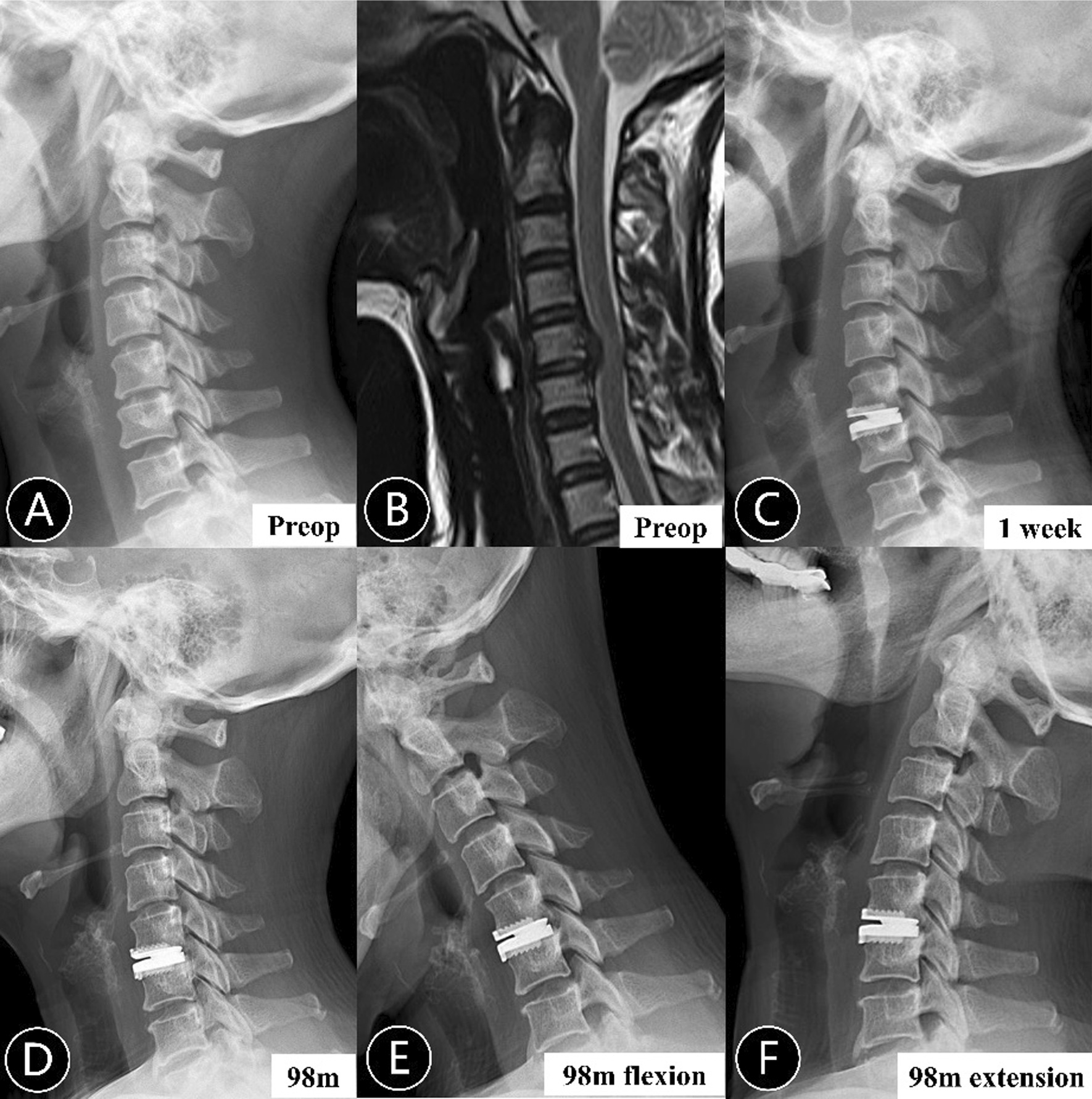
Radiograph of a 50-year-old woman. **A**, **B** Preoperative lateral radiograph and MRI showed decreased intervertebral disc height and compression at C5/6. **C** Lateral radiograph at 1 week after surgery showed a recovery of intervertebral disc height and insufficient endplate coverage at the index level. **D**–**F** X-rays at 98 months follow-up suggested posterior heterotopic ossification with motion preservation at C5/6

In total, 138 patients and 174 arthroplasty levels with at least 2-year follow-up were involved in this retrospective study, including 66 male and 72 female, with a mean age of 43.59 (range, 26–65) years. The median follow-up time was 42 (range, 24–131) months. As shown in Table 1, PHO was detected in 73 segments and 25 motion-restricting PHO identified (Fig. 2). Neither the depth nor the height of prosthesis between groups with and without PHO showed significant differences. No significant differences were noted in the milling angle. The prosthesis-endplate depth ratio in non-PHO group was significantly higher than PHO group (94.92 ± 3.26% vs. 92.13 ± 3.75%, *P* < 0.001). Significantly higher post-operative intervertebral height change was also observed in PHO levels compared to non-PHO levels (2.56 ± 1.04 mm vs. 1.76 ± 0.99 mm, *P* < 0.001). There was no significant difference in the incidence of ABL between the two groups. The patient-reported clinical outcomes including JOA, NDI and VAS showed significant improvement at last follow-up and the scores were comparable between patients with and without PHO (Table 2).

**Table 1:** Comparison of characteristics between levels with and without posterior heterotopic ossification

	non-PHO (*n* = 101)	PHO (*n* = 73)	*P* value
No. of patients, *n*	74	64	–
No. of surgical levels, *n*			
Single-level	59	43	0.949
Level distribution			0.516
C3/4	7	3	
C4/5	17	18	
C5/6	62	44	
C6/7	15	8	
Age, years	43.10 ± 7.94	45.23 ± 8.41	0.110
Sex (M/F)	47/54	38/35	0.472
BMI	23.43 ± 2.87	23.36 ± 2.39	0.884
Blood loss, ml	53.81 ± 28.05	53.84 ± 34.27	0.647
Follow-up, months	48.43 ± 20.62	55.97 ± 28.23	0.276
Milling angle	0.89 ± 3.52	0.83 ± 4.12	0.590
Mean depth of prosthesis, mm	15.31	15.42	0.686
Mean height of prosthesis, mm	5.61	5.63	0.980
Prosthesis-endplate depth ratio, %	94.92 ± 3.26	92.13 ± 3.75	< 0.001*
Post-operative disc height change, mm	1.76 ± 0.99	2.56 ± 1.04	< 0.001*
Anterior bone loss	62	45	0.973

PHO, posterior heterotopic ossification

*Significant difference between two groups

**Table 2:** Clinical outcomes of patients with and without posterior heterotopic ossification

	Patients without PHO (*n* = 74)	Patients with PHO (*n* = 64)	*P* value
JOA score			
Preoperative	12.12 ± 0.83	12.14 ± 0.96	0.809
Last follow-up	16.04 ± 0.73*	15.95 ± 0.67*	0.392
NDI score			
Preoperative	22.39 ± 3.13	22.55 ± 3.51	0.755
Last follow-up	5.64 ± 0.61*	5.66 ± 0.60*	0.649
VAS score			
Preoperative	5.89 ± 0.48	5.86 ± 0.53	0.819
Last follow-up	1.53 ± 0.50*	1.61 ± 0.49*	0.332

PHO, posterior heterotopic ossification; JOA, Japanese Orthopedic Association; NDI, neck disability index; VAS, visual analogue scale

*P < 0.05, compared with pre-operation

### Radiological outcomes

The angular parameters of levels with and without PHO were compared as shown in Table 3. No significant differences of parameters of post-operation and changes during follow-up were observed. At last follow-up, C2–C7 ROM (*P* = 0.035) and ROM at index level (*P* = 0.004) were significantly lower at levels with PHO compared to non-PHO levels.

**Table 3:** Angular parameters of levels with and without posterior heterotopic ossification

	non-PHO (*n* = 101)	PHO (*n* = 73)	*P* value
Post-op			
Cervical lordosis	13.34 ± 9.90	14.32 ± 11.21	0.541
C2–C7 ROM	28.35 ± 11.03	29.35 ± 11.53	0.711
Shell angle	4.99 ± 4.91	4.08 ± 5.18	0.239
FSU angulation	2.40 ± 4.30	3.33 ± 5.23	0.217
ROM at index level	7.10 ± 3.82	7.37 ± 3.66	0.533
Last follow-up			
Cervical lordosis	11.35 ± 8.63	12.85 ± 8.79	0.264
C2–C7 ROM	50.79 ± 13.45	46.33 ± 13.95	0.035*
Shell angle	2.16 ± 5.06	0.97 ± 4.97	0.125
FSU angulation	− 0.47 ± 4.47	0.47 ± 5.06	0.200
ROM at index level	9.12 ± 4.94	7.06 ± 4.51	0.004*
Changes during follow-up			
Cervical lordosis	− 1.99 ± 9.03	− 1.47 ± 9.08	0.712
Shell angle	− 2.82 ± 3.78	− 3.10 ± 4.46	0.659
FSU angulation	− 2.87 ± 3.63	− 2.86 ± 3.63	0.988
Insertion angle	1.37 ± 3.87	1.06 ± 4.01	0.144

PHO, posterior heterotopic ossification; post-op, values at 1 week after surgery; FSU, functional spinal unit angle; ROM, range of motion

*Significant difference between two groups

**Fig. 3:**
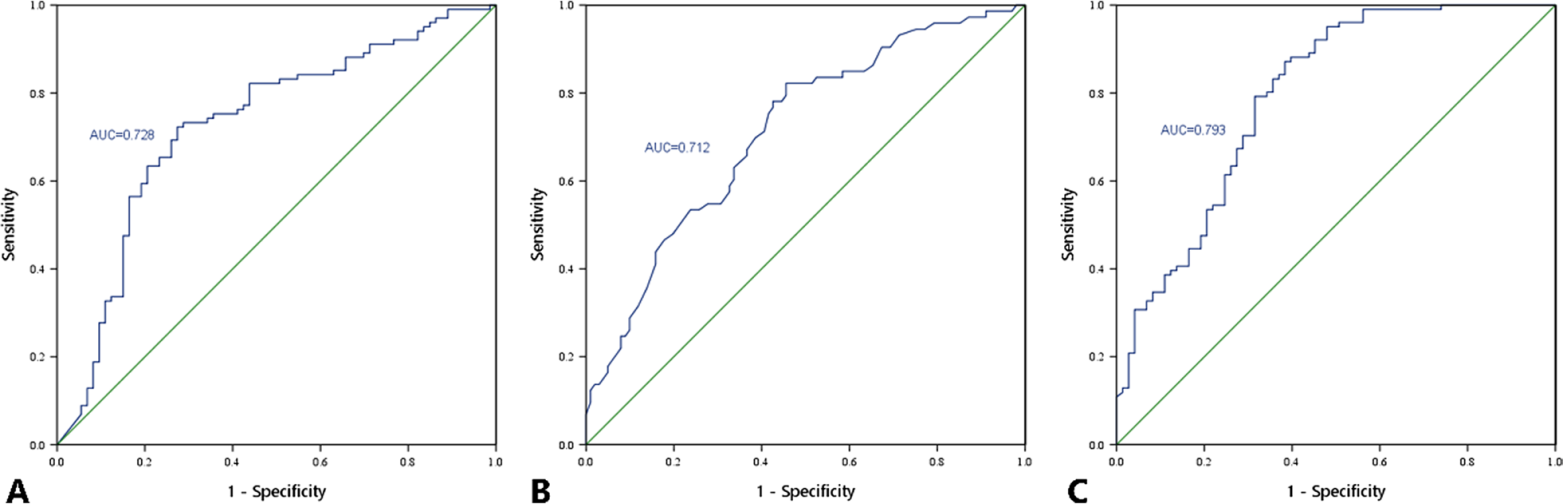
ROC curve of prosthesis-endplate depth ratio (**A**), intervertebral height change (**B**), and CP (**C**) for the prediction of posterior heterotopic ossification. The AUC are 0.728, 0.712, and 0.793, respectively. ROC curve, receiver operating characteristic curve; CP, combined parameter; AUC, area under the curve

Logistic regression analysis confirmed that both the prosthesis-endplate depth ratio (*P* < 0.001, *B* =  − 0.279, OR = 0.757, 95% confidence interval [CI] 0.678–0.844) and post-operative intervertebral height change (*P* < 0.001, *B* = 0.926, OR = 2.523, 95% confidence interval [CI] 1.700–3.746) were predictive factors for the occurrence of PHO (Table 4). According to the logistic regression coefficient, a combined parameter (CP) of two predictive factors was defined as the prosthesis-endplate depth ratio—0.926/0.279 × disc height change, representing the combined effect of endplate coverage and intervertebral height change. A larger CP may denote less biomechanical changes of surgical segment caused by the prosthesis implanting, with optimal endplate coverage and slight change of intervertebral space height. The area under the curve (AUC) of the prosthesis-endplate depth ratio, intervertebral height change and CP were 0.728 (95% confidence interval [CI] 0.650–0.807), 0.712 (95% confidence interval [CI] 0.635–0.789) and 0.793 (95% confidence interval [CI] 0.724–0.863), respectively (Fig. 3). The cut-off values for three factors were 93.77, 1.80, and 84.88, respectively.

**Table 4:** Logistic regression analysis for posterior heterotopic ossification

	*P* value	*B*	OR	95% CI
Prosthesis-endplate depth ratio	< 0.001*	− 0.279	0.757	0.678–0.844
Intervertebral height change	< 0.001*	0.926	2.523	1.700–3.746
Follow-up time	0.644	0.004	1.004	0.988–1.020
Age	0.205	0.029	1.029	0.984–1.076

* Statistical significance

The influences of prosthesis-endplate depth ratio, intervertebral height change and CP were further investigated by dividing the factors into lower and higher groups based on cut-off values. As shown in Table 5, the risk of PHO was significantly increased when the P–E depth ratio < 93.77% (*P* < 0.001, OR = 6.909, 95% confidence interval [CI] 3.521–13.557). The difference of post-operative ROM at the index level between the lower P–E depth ratio group and higher P-E depth ratio was statistically significant (7.77 ± 3.89° vs. 6.73 ± 3.57°, *P* = 0.040). The C2–C7 ROM and ROM at index level were significantly lower in the poor endplate coverage group. Significant larger change in disc insertion angle was observed in the higher P–E ratio group compared with lower P–E ratio group (1.77 ± 3.51° vs. 0.63 ± 4.29°, *P* = 0.039). The incidence of ABL did not show significant difference between the groups. The effect of disc height change is presented in Table 6. The incidence of PHO was significantly higher in the group with disc height change > 1.80 mm (*P* < 0.001, OR = 5.303, 95% confidence interval [CI] 2.592–10.849). At last follow-up, significant better lordosis of cervical spine was noted in the higher disc height change group compared with the other group (13.27 ± 8.37° vs. 9.93 ± 8.90°, *P* = 0.013). No significant difference was observed in the ROM and ABL. The changes of shell angle during follow-up showed significant difference between two groups (− 2.03 ± 3.77° vs. − 3.51 ± 4.16°, *P* = 0.019). As suggested in Table 7, CP < 84.88 was a significant risk factor for PHO (*P* < 0.001, OR = 10.879, 95% confidence interval [CI] 5.142–23.019). Similar to the comparison of different P-E depth ratio groups, significant differences were noted in the ROM at index level post-operatively (*P* = 0.029), ROM of C2–C7 (*P* = 0.018) and ROM at index level (*P* = 0.004) between the two groups. The incidence of ABL was comparable in the two groups.

**Table 5:** Radiographic parameters between poor endplate coverage group and good endplate coverage group

	P–E depth ratio < 93.77 (*n* = 81)	P–E depth ratio ≥ 93.77 (*n* = 93)	*P* value
PHO	53	20	< 0.001*
Motion-restricting PHO	21	4	0.115
Post-op			
Cervical lordosis	13.07 ± 10.31	14.35 ± 10.59	0.621
C2–C7 ROM	28.01 ± 12.12	29.44 ± 10.38	0.229
Shell angle	4.29 ± 5.03	4.88 ± 5.05	0.612
FSU angulation	2.59 ± 4.59	2.96 ± 4.85	0.573
ROM at index level	7.77 ± 3.89	6.73 ± 3.57	0.040*
Last follow-up			
Cervical lordosis	11.11 ± 8.92	12.74 ± 8.48	0.219
C2–C7 ROM	44.94 ± 14.47	52.38 ± 12.25	< 0.001*
Shell angle	1.50 ± 5.43	1.81 ± 4.70	0.687
FSU angulation	− 0.11 ± 4.87	− 0.04 ± 4.64	0.928
ROM at index level	7.31 ± 5.00	9.09 ± 4.61	0.003*
Anterior bone loss	44	63	0.070
Changes during follow-up			
Cervical lordosis	− 1.96 ± 9.10	− 1.61 ± 9.01	0.800
Shell angle	− 2.79 ± 4.19	− 3.07 ± 3.98	0.649
FSU angulation	− 2.70 ± 3.55	− 3.01 ± 3.70	0.575
Insertion angle	0.63 ± 4.29	1.77 ± 3.51	0.039*

*Significant difference between two groups

P-E: prosthesis-endplate; PHO, posterior heterotopic ossification; post-op, values at 1 week after surgery; FSU, functional spinal unit; ROM, range of motion

**Table 6:** Radiographic parameters between low disc height change group and high disc height change group

	Disc height change < 1.80 (*n* = 67)	Disc height change ≥ 1.80 (*n* = 107)	*P* value
PHO	13	60	< 0.001*
Motion-restricting PHO	2	23	0.196
Post-op			
Cervical lordosis	13.19 ± 10.58	14.11 ± 10.40	0.497
ROM C2–C7	30.41 ± 12.02	27.75 ± 10.61	0.131
Shell angle	3.71 ± 4.70	5.17 ± 5.17	0.063
FSU angulation	2.13 ± 4.03	3.20 ± 5.08	0.145
ROM at index level	7.59 ± 3.71	6.98 ± 3.77	0.316
Last follow-up			
Cervical lordosis	9.93 ± 8.90	13.27 ± 8.37	0.013*
ROM C2–C7	49.49 ± 14.06	48.56 ± 13.69	0.664
Shell angle	1.68 ± 4.87	1.66 ± 5.17	0.982
FSU angulation	− 0.66 ± 4.78	0.29 ± 4.69	0.198
ROM at index level	8.61 ± 5.02	8.04 ± 4.77	0.588
Anterior bone loss	40	67	0.701
Changes during follow-up			
Cervical lordosis	− 3.26 ± 9.12	− 0.84 ± 8.89	0.086
Shell angle	− 2.03 ± 3.77	− 3.51 ± 4.16	0.019*
FSU angulation	− 2.79 ± 3.19	− 2.91 ± 3.88	0.817
Insertion angle	0.97 ± 3.77	1.41 ± 4.03	0.462

*Significant difference between two groups

PHO, posterior heterotopic ossification; post-op, values at 1 week after surgery; FSU, functional spinal unit; ROM, range of motion

**Table 7:** Radiographic parameters between low CP group and high CP group

	CP < 84.88 (*n* = 58)	CP ≥ 84.88 (*n* = 116)	*P* value
PHO	45	28	< 0.001*
Motion-restricting PHO	19	6	0.069
Post-op			
Cervical lordosis	12.12 ± 9.91	14.57 ± 10.66	0.211
ROM C2–C7	29.51 ± 11.55	28.40 ± 11.08	0.677
Shell angle	4.70 ± 5.24	4.56 ± 4.95	0.632
FSU angulation	3.20 ± 4.97	2.59 ± 4.60	0.424
ROM at index level	8.07 ± 4.07	6.78 ± 3.52	0.029*
Last follow-up			
Cervical lordosis	11.92 ± 8.70	12.01 ± 8.74	0.945
ROM C2–C7	44.83 ± 13.96	50.96 ± 13.31	0.018*
Shell angle	1.83 ± 5.78	1.58 ± 4.66	0.759
FSU angulation	0.69 ± 4.75	− 0.46 ± 4.70	0.134
ROM at index level	6.89 ± 4.70	8.94 ± 4.82	0.004*
Anterior bone loss	32	75	0.226
Changes during follow-up			
Cervical lordosis	− 0.20 ± 8.70	− 2.56 ± 9.12	0.104
Shell angle	− 2.87 ± 3.96	− 2.98 ± 4.14	0.874
FSU angulation	− 2.51 ± 3.61	− 3.04 ± 3.63	0.263
Insertion angle	0.85 ± 4.64	1.43 ± 3.51	0.260

*Significant difference between two groups

CP, combined parameter; PHO, posterior heterotopic ossification; post-op, values at 1 week after surgery; FSU, functional spinal unit; ROM, range of motion

## Discussion

In the treatment of cervical radiculopathy and myelopathy, CDR is introduced to reconstruct the physiological motion of diseased segment. The formation of HO after CDR is one of the major obstacles in the development of non-fusion technique of cervical surgery. However, the detailed mechanism of HO is still controversial. The change of biomechanical environment is considered a main contributing factor. HO formation is postulated to be a self-defence mechanism responding to the non-physiological biomechanics of cervical spine after CDR, which is influenced by the endplate coverage and disc height [14, 25]. The present study focused on the effects of endplate coverage, intervertebral height change and their combined effect on HO formation. Previous study has suggested an evidently higher incidence of HO in the posterior disc space and different risk factors for HO in the anterior and posterior disc space [26]. Therefore, only PHO was taken into consideration in this study due to the anterior-limiting design of Prestige-LP disc. The results suggested that the occurrence of PHO did not affect the patient-reported outcomes. This is consistent with prior studies, suggesting that the satisfactory outcomes of CDR mainly depend on adequate surgical decompression [10, 27, 28]. Both prosthesis-endplate depth ratio and intervertebral height change were potential risk factors for the development of PHO after CDR. The risk of PHO significantly increased when the prosthesis-endplate depth ratio was less than 93.8% or the change of intervertebral disc height after surgery was large than 1.80 mm.

Due to the irregularity of cervical endplate morphology, the mismatch between the prosthesis and endplate is usually unavoidable [29]. Thaler et al. [30] reported that 43.7% of Bryan and ProDisc-C, 60.4% of Discover, and 100% of Prestige footprints did not match the endplate regarding anterior–posterior diameters. Insufficient endplate coverage is thought to lead to the occurrence of HO. Tu et al. [12] retrospectively evaluated the perfectness of carpentry for each arthroplasty level with Bryan disc, which defined the inadequate endplate coverage and shell kyphosis of index level as suboptimal group. They found that the suboptimal carpentry group had significantly more high-grade HO (≥ Grade 2) than the optimal carpentry group. Zeng et al. [15] reported that the inadequate width and depth of the Prestige-LP relative to the endplate are likely to induce the formation of HO. Xu et al. [17] concluded that HO was more prone to occur when the uncovered sagittal distance ≥ 2.5 mm. Another study by Guo et al. [16] also revealed that the HO occurrence was significantly related with footprint matching degree using three-dimensional computed tomographic images. In this study, the poor endplate coverage group with prosthesis-endplate depth ratio < 93.77% suggested a significantly higher incidence of PHO. However, the motion-restricting PHO rates were not significantly different between the better and poor endplate coverage group. This is consistent with the study of Kim et al. [18] that endplate coverage was not significantly related to the ROM-limiting HO, which probably indicating a distinct mechanism for high-grade HO and need to be further investigated. They also found that the increased segmental ROM was related to the formation of high-grade HO. However, a meta-analysis of 1674 patients found that neither HO nor the high-grade HO was associated with the segmental ROM [31]. Although our study found a significantly larger segmental ROM after surgery and smaller change in disc insertion angle in the poor endplate coverage group, we believe the minor differences between the two groups not reaching clinical significance. Tian et al. [24] found that patients with progressed HO showed greater change in disc insertion angle by retrospectively reviewing patients who underwent CDR with Bryan. The use of different types of implants may explain the discrepancy. A finite element analysis found that the endplate stress was much higher in the models with Prestige-LP and ProDisc-C, compared with the model with Bryan [32]. That is, the biomechanics of the artificial itself also have an impact on HO formation and further investigations with large patient sample and different prosthesis are needed [16, 26, 28].

HO formation is postulated to compensating for the non-uniform stress distribution, which is one of the mechanical elements associated with the bone remodelling after CDR [13]. HO may occur when the implanted artificial disc fails to restore normal loading patterns in the surgical segment. Previous studies suggested a significantly lower incidence of HO after CDR with ProDisc Vivo disc, whose design had the potential benefits of matching the anatomical feature of vertebral endplate and reducing the violation of endplate [33, 34]. Palissery et al. [35] found that the use of smaller size artificial discs caused localized stress concentration in the implant-bony endplate interface while a well-fitting prosthesis contributed to a more physiological and uniform stress distribution through finite element analysis. Ganbat et al. [13] developed a three-dimensional finite element model simulating a bone adaptation process after CDR and found that most of the HO developed on the vertebral endplates uncovered by the prosthesis footplate under compressive force. Interestingly, HO formation itself reduced the peak values and total values of the strain energy of the endplate, which is more obvious in the posterior disc region without footplate coverage. Since an artificial disc may not cover the whole vertebral endplate because of surgical restriction, the prosthesis-endplate depth ratio less than 93.8% should be avoided according to our results.

Insufficient endplate coverage leads to non-uniform stress pattern in the margin of prosthesis, which may be exacerbated by larger intervertebral height change post-operatively. Although increase in intervertebral height is conducive to the neurological decompression, inappropriate disc height increment may alter the segmental biomechanical environment and increase the stress of prosthesis-endplate interface [14, 19]. The present study showed that intervertebral disc height change was significantly higher in the PHO group, and the most suitable cut-off for disc height change to predict HO is 1.80 mm. Wang et al. [21] found that the degree of distraction of index level was significantly larger in patients with HO following CDR. Another study by Kim et al. [18] also identified significantly higher differences in height in the high-grade HO group than in the low-grade HO group. Our findings suggested that larger disc height distraction was associated with better cervical lordosis at last follow-up. The change of shell angle during follow-up in the large disc height change group was significantly higher, indicating that the formation of PHO might have been adapted to the change of biomechanical environment after CDR [14, 24]. The combined effects of endplate coverage and intervertebral height were further investigated, and an increased AUC for predicting PHO was suggested in this study. We found that ROM at index level post-operatively was significantly larger in the low CP group, however, limited clinical implication due to the small differences. In addition, uneven loading force in the bone-implant interface of cervical artificial disc was also shown to be related to ABL after CDR. Chen et al. [36] revealed that increasing the shell angle may increase the incidence of ABL after CDR because of the decreased loading force in anterior space. This study suggested that ABL seemed not related to the biomechanics caused by changes of endplate coverage and intervertebral height, which probably contributed more to the posterior biomechanical environment. Therefore, detailed stress distribution caused by different endplate coverage and intervertebral height change is needed further investigation.

The limitations of the present study deserve consideration. First, the retrospective nature presented inherent weakness. Second, the minimum of 2-year follow-up was relatively short and the incidence of HO might be underestimated. Third, although the radiological parameters were collected according to the previously published literature, it should be acknowledged that the inherent potential of error in radiographic imaging may be a major limitation of this study. Fourth, the single institution study and prosthesis type limited the generalizability of the results. Thus, multicentre, prospective studies with long-term follow-up and various disc types are needed. Despite these limitations, this is the first study to focus on the effect of segmental biomechanics due to variations of endplate coverage and interverbal disc height after CDR on HO formation through quantitative analysis.

## Conclusions

Inadequate endplate coverage and excessive change of intervertebral height are both critical risk factors for the PHO formation after CDR while have no effect on ABL. Endplate coverage less than 93.8% or intervertebral height change more than 1.8 mm would increase the risk of PHO. The combination of these two factors may exacerbate the non-uniform distribution of stress in the bone-implant interface and promote HO formation. At least one of the two issues should be avoided during surgery to prevent the occurrence of HO.

## Data Availability

The datasets used in this study are available from the corresponding author on reasonable request.

## Abbreviations

ACDF: Anterior cervical discectomy and fusion
CDR: Cervical disc replacement
ASD: Adjacent segment disease
HO: Heterotopic ossification
JOA: Japanese Orthopaedic Association
NDI: Neck Disability Index
VAS: Visual analogue scale
ABL: Anterior bone loss
PHO: Posterior heterotopic ossification
CT: Computed tomography
ROM: Range of motion
FSU: Functional spinal unit
ROC: Receiver operating characteristic
AUC: Area under the curve
